# Effect of applying carbodiimide combined with a two-step self-etch adhesive durability

**DOI:** 10.1186/s12903-024-04415-2

**Published:** 2024-06-07

**Authors:** Lin Tang, Yi Zhang, Yuhua Liu, Xiaoying Chen, Yuke Li, Lingli Zhu

**Affiliations:** 1 Department of Prosthodontics, Peking University School and Hospital of Stomatology & National Center of Stomatology & National Clinical Research Center for Oral Diseases & National Engineering Research Center of Oral Biomaterials and Digital Medical Devices & Beijing Key Laboratory of Digital Stomatology & National Health Commission Key Laboratory of Digital Technology of Stomatology, Beijing, China; 2 Department of General Dentistry II, Peking University School and Hospital of Stomatology & National Center of Stomatology & National Clinical Research Center for Oral Diseases & National Engineering Research Center of Oral Biomaterials and Digital Medical Devices & Beijing Key Laboratory of Digital Stomatology & National Health Commission Key Laboratory of Digital Technology of Stomatology, Beijing, China; 3 Department of Stomatology, Shanghai Fourth People’s Hospital, School of Medicine, Tongji University, Shanghai, China; 4 Department of Prosthodontics, Peking University School and Hospital of Stomatology, 22# Zhongguancun South Avenue, Haidian District, Beijing, 100081 China

**Keywords:** Aging, Carbodiimide, Dentin bonding agent, Shear strength

## Abstract

**Background:**

This study investigated the effect of carbodiimide (EDC) combined with Clearfil SE self-etch adhesive on the shear bond strength (SBS), crosslinking degree, denaturation temperature, and enzyme activity of dentin in vitro.

**Materials and methods:**

Collected human sound third molars were randomly divided into different groups with or without EDC treatment (0.01–1 M). The specimens (*n* = 16)were stored for 24 h (immediate) or 12 months (aging) before testing the SBS. Fine dentin powder was obtained and treated with the same solutions. Then the crosslinking degree, denaturation temperature (Td), and enzyme activity were tested. Statistical analysis was performed using a one-way analysis of variance (ANOVA) to compare the differences of data between groups (*α* = 0.05).

**Results:**

There was a significant drop in immediate SBS and more adhesive fracture of 1.0 M EDC group, while there were no significant differences among the other groups. SEM showed a homogeneous interface under all treatments. After 12 months of aging, the SBS significantly decreased. Less decreases of SBS in the 0.3 and 0.5 M groups were found. Due to thermal and enzymatical properties consideration, the 0.3 and 0.5 M treatments also showed higher cross-link degree and Td with lower enzyme activity.

**Conclusion:**

0.3 and 0.5 M EDC may be favorable for delaying the aging of self-etch bond strength for 12 months. But it is still needed thoroughly study.

## Introduction

Dentin bonding and aging have been a recent research focus. Dentin collagen fibrils, as the primary constituents of the hybrid layer in dentin bonding, play an important role in these processes, and their rate of degradation significantly determines the long-term effectiveness of dentin bonding [1, 2]. Preserving their integrity at the bonding interface is important during the aging process [3]. Theoretically, the resistance of collagen fibrils to aging can be enhanced by improving their inherent resistance to hydrolysis and enzymatic degradation or by inhibiting the activity of endogenous and exogenous proteases [4]. Physicochemical crosslinking technique is an important strategy to this end [5, 6] and enhances the stability and durability of the bonding interface [4].

Carbodiimide hydrochloride [1-ethyl-3-(3-dimethylaminopropyl carbodiimide), EDC] is a synthetic crosslinking agent with good biocompatibility [7] and excellent crosslinking properties [8] that is widely used in collagen modification [9], biomimetic mineralization [10], and bone tissue regeneration [11]. It nonspecifically activates the carboxylic acid groups of glutamic and aspartic acids in proteins to form amide crosslinks, irreversibly altering the three-dimensional structure of the peptides and inactivating the catalytic site of proteases, effectively suppressing enzymatic degradation. Furthermore, it promotes the crosslinking of dentin collagen fibrils, which enhances the mechanical properties and enzymatic resistance of the dentin matrix to increase the durability of resin–dentin bonds [8].

The total-etch bonding adhesive such as Single Bond 2 is commonly to research the use of carbodiimide to slow dentin bond aging [12, 13]. However, it has limitations such as over etching of dentin, high technical sensitivity for wet bonding, and postoperative sensitivity [1, 14]. Clearfil SE Bond, a self-etch adhesive, known for its ease of operation and reduced postoperative sensitivity, is widely used for dentin bonding in practice [15]. However, there is little research on the role of carbodiimide in self-etch bonding strategies, and the results of studies that have investigated it are not conclusive. For example, there is no consensus on the concentration and time settings of carbodiimide treatment [16]. The mechanism of self-etch dentin bonding, which retains the smear layer to form a hybrid layer, is significantly different from total-etch dentin bonding [15]. Whether this bonding mechanism impacts the effect of carbodiimide on bond aging is worthy of in-depth exploration. Therefore, this study examined the effectiveness of carbodiimide in self-etch dentin bonding and explored the possibility of using carbodiimide to resist bond aging via its effects on matrix metalloproteinases and the thermal properties of dentin collagen fibrils. The null hypothesis tested were that the application of carbodiimide combined with a self-etch adhesive does not (1) preserve dentin bond strength, (2) enhance dentin collagen thermal stability and (3) inactive enzyme activity.

## Materials and methods

Totally 190 human third molars were collected from 18- to 40-year-old patients at a protocol approved by the Biomedical Ethics Committee of Peking University School and Hospital of Stomatology. All subjects gave their informed consent for inclusion before they participated in the study. The study was conducted in accordance with the Declaration of Helsinki. The obtained teeth were intact without caries, restorations, severe periodontal diseases, apparent wear, or cracks. Attached periodontal tissues were removed with a scalpel after extraction, and the teeth were immediately stored at 4ºC in 0.9% NaCl containing 0.5% (w/v) chloramine-T to prevent bacterial growth and utilized within one month.

### Immediate and aging shear bond strength test for carbodiimide’s effect on dentin bond aging

Every collected teeth had their occlusal enamel removed using a diamond bur (Dia-Burs, MANI, Japan) under water irrigation creative1-2 dentin discs with a thickness of 1mm. The discs were embedded in acrylic resin. The exposed mid-coronal dentin surfaces were grinded sequentially using 200, 400 and 600-grit SiC papers (Panda, Beijing East New Grinding Tools Co., Ltd., China) under running water for 30 s. All the specimens (dentin discs) were randomly divided into thirteen groups according to the surface treatment (Table 1).

**Table 1:** The grouping of the shear bond strength test

Time	Treatment^a^						
Immediate (24 h)	Untreated	80% ethanol	0.01M EDC	0.1M EDC	0.3M EDC	0.5M EDC	1M EDC
Aging (12 month)	Untreated	80% ethanol	0.01M EDC	0.1M EDC	0.3M EDC	0.5M EDC	

^a^Treatment was applied after Clearfil SE Bond primer

Untreated/Control groups: Bonding procedures were accomplished following the instruction of Clearfil SE Bond (Kuraray, Osaka, Japan). Resin cylinders with a height of 3 mm using Clearfil AP-X composite resin (Kuraray, Osaka, Japan) were built up in 1 mm thick increment. Each increment was light cured with a light-emitting diode curing unit (output, 1200 mW/cm[2]) for 20 s. A PTFE (polytetrafluoroethylene) split mold was used and the radius of dentin bonding surface was designed to be 1 mm.

Ethanol and carbodiimide treatment groups: Clearfil SE Bond primer was applied on the dentin surface for 20 s and air dried. Ethanol solvent or freshly prepared carbodiimide ethanol solutions at concentrations of 0.01 M, 0.1 M, 0.3 M, 0.5 M, 1 M were evenly applied on the dentin surface and kept moist for 60 s. Excess treatment solution was removed by small sponges. After that bond solution was applied, a uniform film was made by gentle air blow and light cured for 10 s. Composite resin was built the same as described in the untreated groups.

Sixteen specimens were made for each group (*n* = 16, totally 13 groups) and stored in 0.9% saline solution containing 2% chloramine-T at 37°C for 24 h (immediate groups) or 12 months (aging groups) respectively.

The specimens were fixed and mounted in the apparatus and the SBS test was performed in line with the standard ISO/TS 11405 − 2003 using a universal test machine (EZ-L, Shimadzu, Kyoto, Japan) with a crosshead speed of 1 mm/min. The shear force value (Force, F) was recorded at the point of loading fracture. The shear bond strength was calculated as the following formula: SBS = F/πr².

The fractured interfaces of all specimens were examined under a stereomicroscope (SMZ 1500, Nikon, Tokyo, Japan) at 10× magnification. The failure modes were categorized as adhesive failure (AF) that occurs at the bonding interface, cohesive failure (CF) occurs within the resin or dentin, and mixed failure (MF) involving a combination of both.

### Scanning electron microscopy (SEM) observation of the bonding interfaces

The specimens (*n* = 4) for each immediate group dealt as above were sectioned, dried and sputter-coated with platinum-palladium alloy. Microscopic observation (2000x) of the bonding interface was conducted using a cold field emission scanning electron microscope (S-4800, Hitachi, Tokyo, Japan).

### Cross-linking degree evaluation

After removal of the tooth enamel, pulp tissue, and root, all dentin pieces were obtained and dehydrated in acetone for 5 min, then triturated to fine powder with a stainless-steel mixer mill (Moderl MM400, Retsch, Newtown, PA, USA) in a liquid nitrogen atmosphere for 20 min before two-sieve screening (mesh sizes: 15 and 30 µm).

The demineralized dentin powder was equally divided to six groups by weight. Md (mineral dentin powder) group: sound dentin powder without treatment. Md + 0.3M EDC/0.5M EDC groups: 800 µL of 0.3 M or 0.5 M EDC solution was respectively added to a centrifuge tube containing mineral dentin powder, stirred continuously for 60 s. CSp (Clearfil SE Bond primer) Group: sufficient amount of Clearfil SE Bond primer was applied to dentin powder for 20 s. CSp + 0.3M EDC/0.5M EDC groups: 800 µL of 0.3 M or 0.5 M EDC solution was respectively added to a centrifuge tube containing primer demineralized dentin powder, stirred continuously for 60 s. All the samples were thoroughly diluted, washed, centrifuged at 1500 rpm for 10 min, discarded the supernatant, and the dilution and centrifugation steps were repeated three times, then placed in a desiccator containing color-changing silica gel and dried overnight.

The cross-linking degrees of samples from different groups were determined through the reaction of ninhydrin (2,2-dihydroxy-1,3-indanedione) with the primary amine groups of collagens according to the absorption spectroscopy method as previous study described [13].

### Denaturation temperature

The denaturation temperature of dentin powder from each group as described above were analyzed with a differential scanning calorimeter (DSC, Q20 TA Instruments, New Castle, DE, USA). The thermal denaturation temperature of each sample was obtained.

### Measurement of enzyme activity

Enzymatic activity of the dentin powder from each group as described above was assessed using the EnzChek gelatinase/collagenase assay kit (Molecular Probes, Eugene, OR) with a microplate reader (EnSpire™ Multimode Plate Reader, PerkinElmer, Inc., Waltham MA, US). The principle of the enzyme detection was briefly illustrated in Fig. 5a [17]. Excitation wavelength was set at 485 ± 10 nm, and detection wavelength was 530 ± 15 nm. The relative fluorescence units (RFU) of the samples from each group were calculated.

### Statistical analysis

A one-way analysis of variance (ANOVA) was used to analyze the differences in shear bond strength, cross-linking degrees, denaturation temperatures and relative fluorescence units between groups using IBM SPSS Statistics (version 20.0, IBM SPSS, Chicago, IL, USA). LSD method was used for multiple comparisons. Furthermore, the frequency of the failure modes was analyzed using a chi-square test and Fisher’s exact test. The significance level was set at 0.05 for all statistical comparisons.

## Results

### Immediate shear bond strength and failure modes

**Fig. 1:**
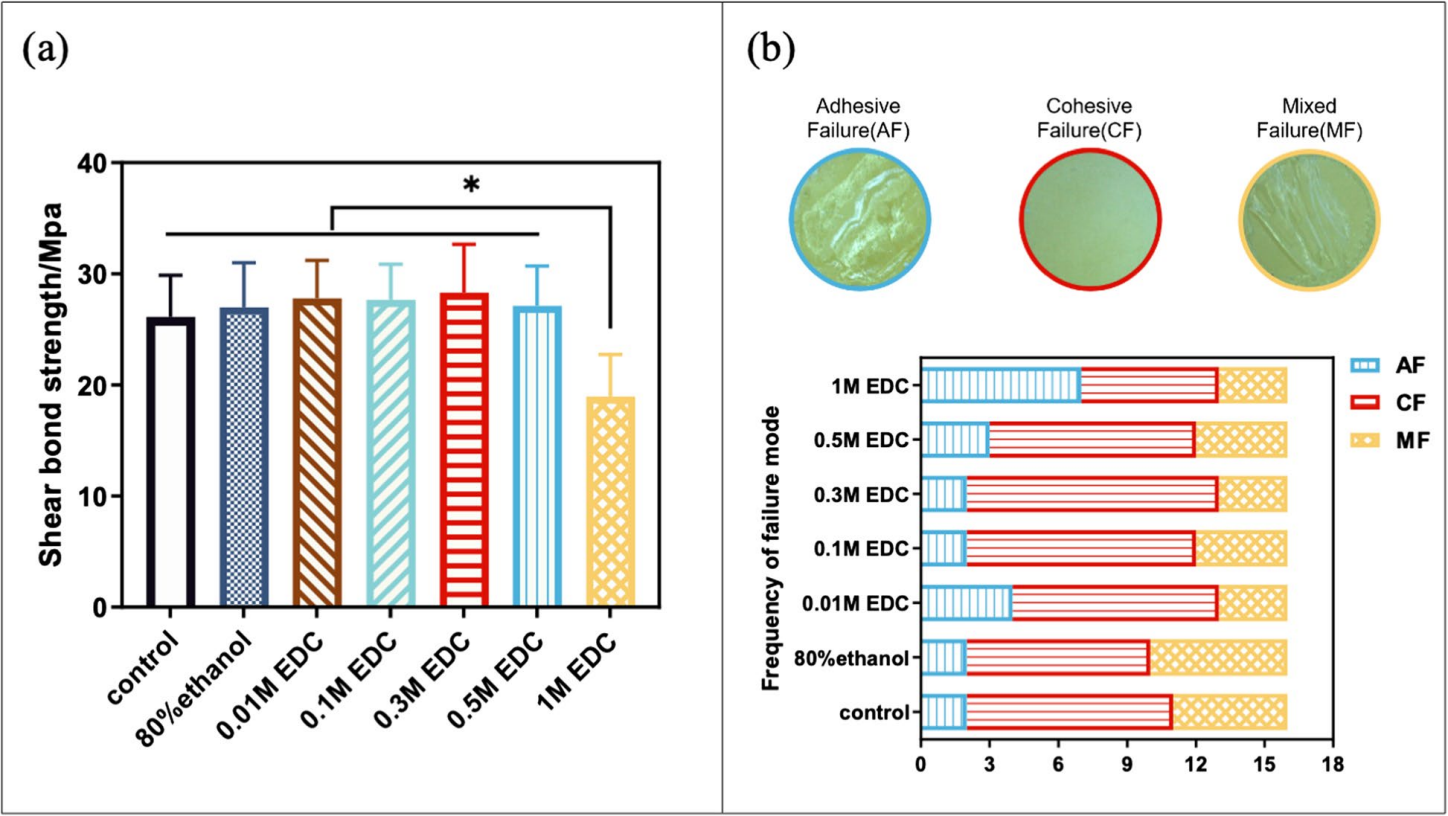
**a** The shear bond strength with Clearfil SE Bond of different dentin surface treatments. The asterisk indicates a significant difference in SBS between groups (*P* < 0.05). The SBS of 1 M EDC treated group were significantly lower than the corresponding data obtained from other groups. **b** The representative micrographs of the fractured interfaces examined by stereomicroscope were classified as adhesive failure (AF), cohesive failure (CF) and mixed failure (MF), respectively. Orig. mag ×10. The failure mode frequency of the immediate SBS test for different treatment groups were illustrated in the chart. No significant difference was found among groups

The mean values of immediate shear bond strength using Clearfil SE Bond adhesive ranged from 20.24 to 29.56 MPa. The SBS significantly decreased in the 1M EDC group (*P* < 0.05). The values of SBS in groups treated with 80% ethanol solvent and EDC at concentrations of 0.01 M, 0.1 M, 0.3 M,0.5 M were slightly higher than that in the control group, but the differences among these groups were not statistically significant (*P* > 0.05) (Fig. 1a).

The main failure mode in all groups was cohesive failure, followed by mixed failure mode. The proportion of adhesive failure mode in 1M EDC group was higher than that of other groups. The chi-squared test of the failure mode frequency of the SBS test specimens indicated that there was no significant difference among groups (*P* = 0.576 > 0.05) (Fig. 1b).

### SEM of bonding interfaces

**Fig. 2:**
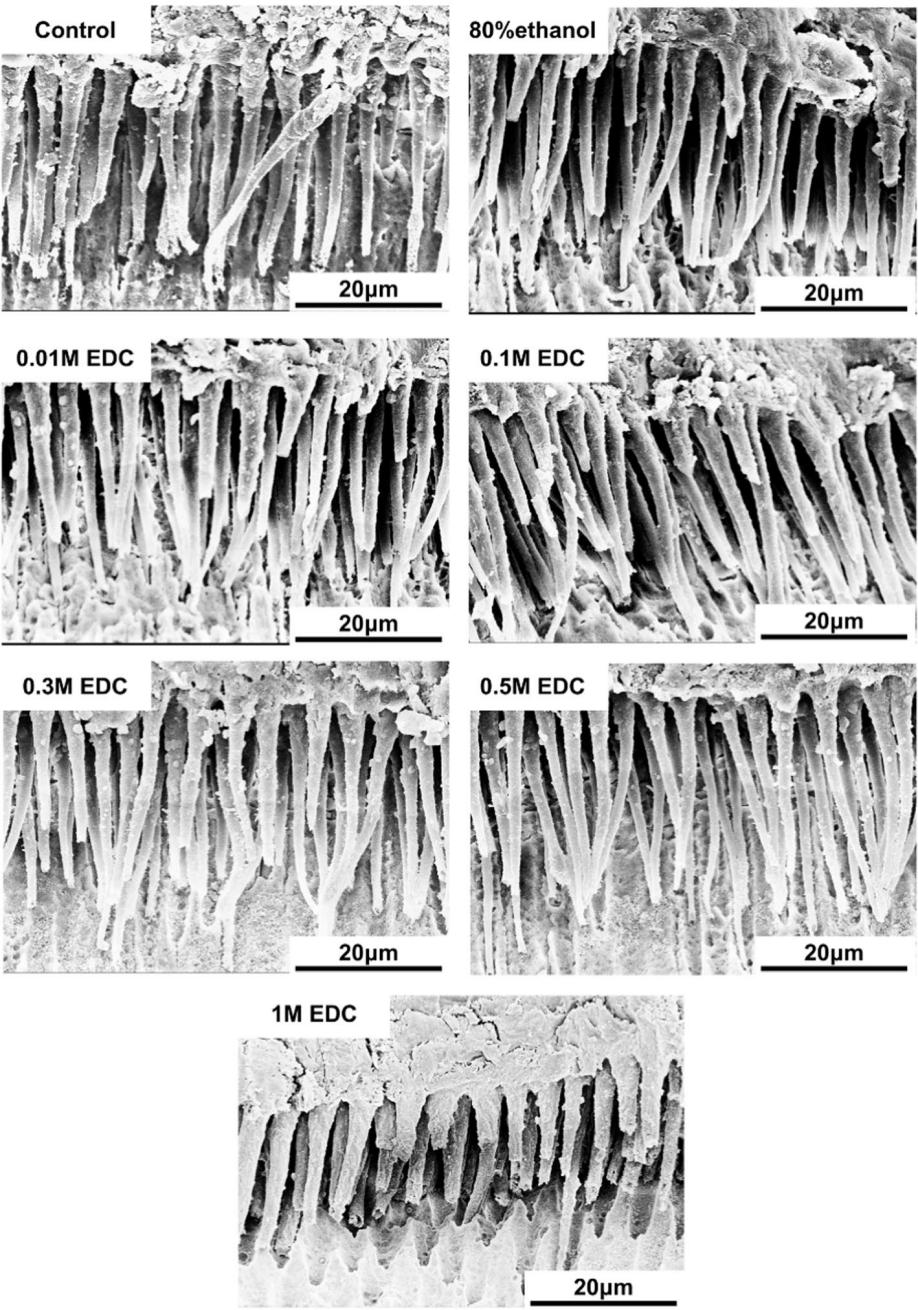
Representative SEM micrographs of the resin-dentin interface obtained after 24 h storage in 0.9% saline solution. Insufficient infiltration of the adhesive and short, conical resin tags can be observed in the specimen treated with 1 M EDC solution. Uniform, continuous hybrid layers and long, well-formed resin tags with lateral branches can be found in control group, 80% ethanol, 0.01 M, 0.1 M, 0.3 M and 0.5 M EDC treated groups

Representative SEM micrographs of the resin-dentin interfaces were obtained. The resin tags at the bonding interface in the control group exhibited a well-formed morphology with a uniform and continuous hybrid layer. Intact and complete hybrid layers and dentine tubules lateral branches infiltrated by long resin tags can also be found in 80% ethanol, 0.01 M, 0.1 M, 0.3 M and 0.5 M EDC treated groups. Shorter resin tags were seen within the resin-dentin joints in the 1M EDC group specimen (Fig. 2).

### Shear bond strength and failure modes after aging

**Fig. 3:**
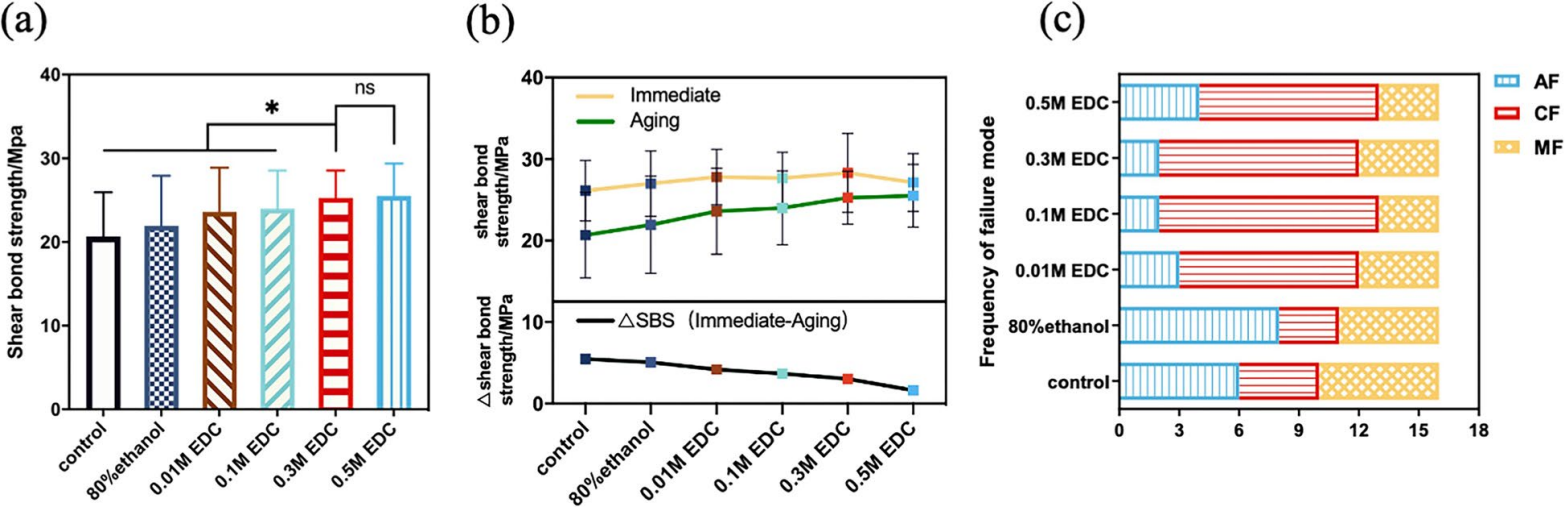
**a** The shear bond strength of different dentin surface treatments after 12 months aging. The asterisk indicates a significant difference between groups. 0.3 M and 0.5 M EDC treatment significantly preserve dentin bond strength compare with the other groups (*P* < 0.05). **b** The upper part of the line chart compares the immediate and aged shear bond strength within groups. The lower indicates the decreases of SBS after aging for each group. **c** The failure mode frequency of the SBS test for different treatment groups after 12 months aging

After 12 months of aging, the shear bonding strength has declined to a certain extent. The results showed that time and treatment method significantly affected dentin shear strength. The specimen treated with 0.3 M and 0.5 M EDC had significantly higher SBS after aging compared to the other groups (*P *< 0.05) (Fig. 3a). There were significant differences between the immediate and aged SBS within groups. The decreases percentage of bonding strength differed from groups (control group: 27.90%, 80% ethanol group: 25.74%, 0.01 M EDC group: 18.78%, 0.1M group: 17.09%, 0.3 M EDC: 14.56%, 0.5 M EDC group: 10.17%) as shown in Fig. 3b.

The proportions of adhesive failure and mixed failure modes in the control group and ethanol treated group increased, reaching 75% and 81.25% respectively. Whereas the cohesive failure remained the dominant failure mode in the EDC treated groups (Fig. 3c).

### Cross-linking degree and denaturation temperature

**Fig. 4:**
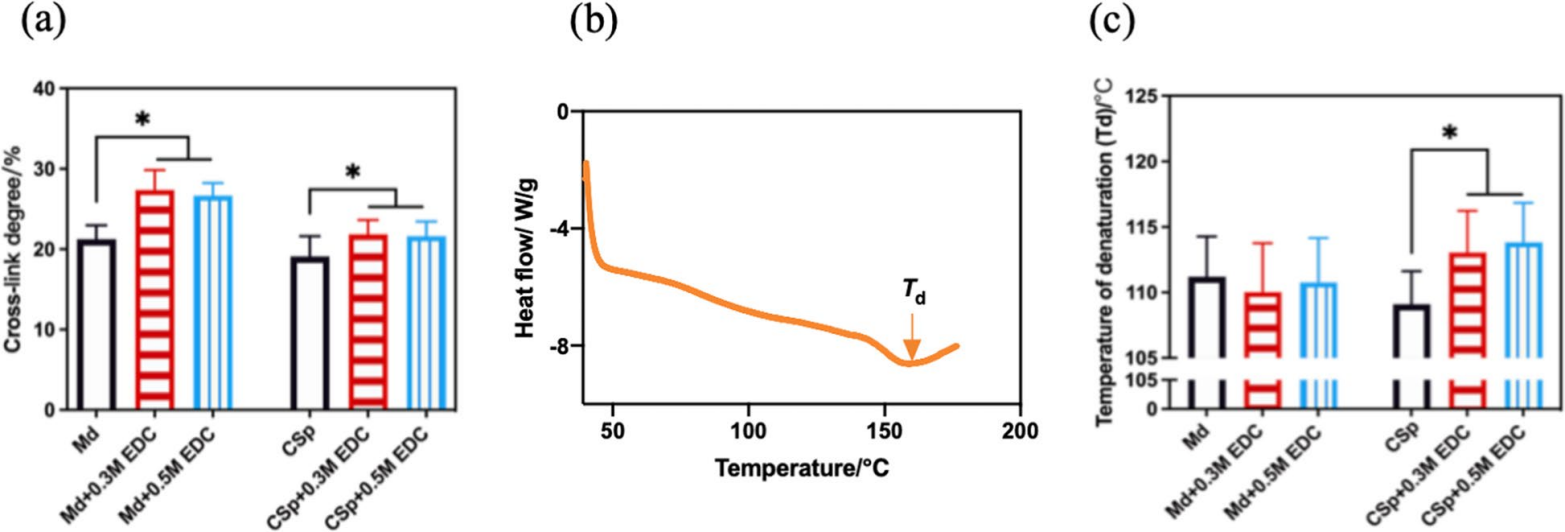
**a** The cross-linking degrees of mineral dentin matrix and demineralized dentin matrix treated with and without EDC solutions. **b** The DSC thermogram of the dentin powder. The endothermal peak pointed by arrow as Td in the chart illustrates the denaturation temperature of the specimen. **c** The denaturation temperature of mineral dentin matrix and demineralized dentin matrix treated with and without EDC solutions. The asterisk indicates a significant difference between groups (*P* < 0.05)

The degrees of cross-linking were shown in Fig. 4a. After treated with 0.3M and 0.5M EDC, the cross-linking degrees of mineral dentin matrix and demineralized dentin matrix significantly increased (*P* < 0.05). The denaturation temperature of demineralized dentin matrix significantly increased after 0.3M and 0.5M EDC treatment (*P* < 0.05). There was no significant difference of the denaturation temperature between mineral dentin matrix groups (*P* > 0.05) (Fig. 4b, c).

### Enzyme activity

**Fig. 5:**
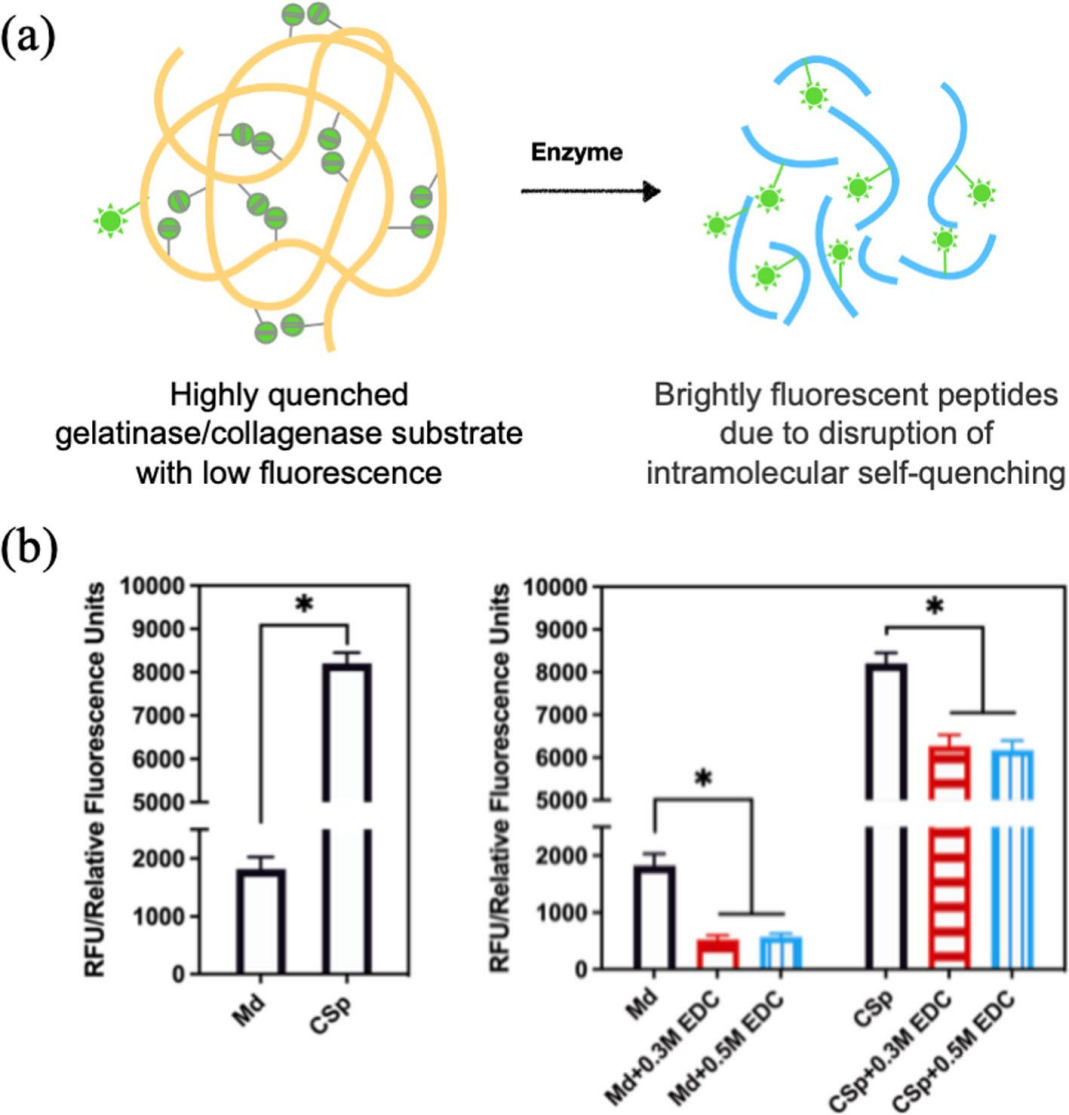
**a **Schematic illustration of the principle of enzyme detection via the disruption of intramolecular self-quenching adapted from technical reference provided by manufacturer [17]. **b** The relative fluorescence units of mineral dentin matrix and demineralized dentin matrix treated with and without EDC solutions. The asterisk indicates a significant difference between groups (*P* < 0.05)

The results of enzymatic activity tested by EnzChek gelatinase/collagenase assay kit were shown in the Fig. 5b, indicated a lower enzymatic activity in the mineral dentin matrix. After treatment with Clearfil SE Bond, the enzymatic activity of dentin matrix significantly increased. 0.3M and 0.5 M EDC treatment significantly reduced the enzymatic activity in the CSp groups.

## Discussion

This study used a two-step self-etch adhesive (Clearfil SE-Bond) to evaluate the effects of different carbodiimide concentrations on dentin bonding strength and aging. Previous studies of the use of carbodiimide in dentin bonding have mostly used the total-etch technique [18]. Scheffel et al. found that the crosslinking efficiency of carbodiimide is concentration-dependent [19]. However, there is no consensus on the optimal concentration of carbodiimide treatment to improve dentin bonding, and few studies have investigated how carbodiimide crosslinking modification may influence self-etch adhesive [20, 21]. Therefore, we evaluated the influence of carbodiimide at various concentrations from a high of 1 M to a low of 0.01 M on the immediate bonding strength of self-etch dentin. Ethanol was chosen over water due to its higher vapor pressure, aiding in residual water removal and preventing phase separation as described in our previous study [10]. Besides, Single Bond 2 is a water–ethanol solvent system. Treatment with concentrations no more than 0.5 M did not reduce the immediate shear bond strength, while 1 M carbodiimide significantly decreased it. Microscopy revealed that the structure of bonding interfaces in groups treated with 0.01, 0.1, 0.3, and 0.5 M carbodiimide were more uniform, continuous, and consistent. The branches of dentinal tubules were filled with adhesive resin, indicating that the adhesive effectively penetrated the collagen fibrils and formed a hybrid layer. By contrast, the resin tags in the 1 M carbodiimide group were relatively short. Although previous studies have not explicitly correlated resin tag length with dentin bonding strength [22, 23], the significant decrease in bonding strength in this group could be attributable to the potential formation of carbodiimide crystal aggregates on the treated dentin surface with the evaporation of the solvent, hindering the penetration of adhesive resin.

To simulate the bond aging process, the shear bond strengths of samples were tested after 12 months of water-storage aging. This showed a significant decline in the shear bond strength in all groups after aging and the rate of decrease was concentration-dependent. The shear bond strengths of samples in the control and ethanol solvent groups showed more pronounced declines with more adhesive failures. This was attributed to the increased activity of endogenous proteases in the dentin matrix due to the priming and bonding procedures of the self-etch bonding agent, leading to the gradual degradation of collagen fibrils within the hybrid layer [24, 25]. Overall, samples treated with 0.3 and 0.5 M carbodiimide had more stable bonding strengths after aging compared to controls. Hence, the first null hypothesis has to be rejected. This is similar to the results of Mazzoni et al., who suggested that 0.3 M carbodiimide has a significant maintenance effect on micro-tensile bonding strength using self-etch adhesive after aging for 1 year [20]. Zhang et al. found that 0.5 M EDC crosslinking treatment maintained fatigue crack growth resistance for 6 months after bonding [26]. Singh et al. found that 0.3 M carbodiimide pretreatment of the dentin surface before applying a one-step self-etch adhesive significantly preserved the resin–dentin bond over a 6-month storage period [16]. In our study, the acidic monomers in the primer mildly demineralized the interface of the dentin matrix, which facilitates carbodiimide-induced crosslinking of the collagen fibrils and improves resistance to dentin bond aging [21].

Enzymatic degradation of the collagen matrix by endogenous proteases plays a significant role in the aging of the dentin bonding interface [8]. Weak fluorescence activity of gelatinase and collagenase was detected in the mineralized dentin powder, suggesting the presence of basic enzymatic activity in the dentin matrix itself. When simulating clinical bonding using Clearfil SE Bond, the dentin powder after priming showed strong fluorescence activity, indicating a significant increase in gelatinase/collagenase activity. This is consistent with studies showing that although endogenous proteases exist in an inactivated form in mineralized dentin matrix, the acidic components (pH 1.5–2.7) in self-etch adhesives can activate the proteases, affecting the durability and stability of dentin bonding [8, 27].

The degree of crosslinking and denaturation temperature are indicators of collagen thermal stability and intuitively reflect the performance of crosslinking agents. In our study, 0.3 and 0.5 M carbodiimide treatment improved the stability of the dentin matrix and the second null hypothesis was rejected. Carbodiimides can activate free carboxyl groups for direct reaction with amine groups via stable amide bond formation, resulting in covalent cross-links [28]. In this way, they increase the mechanical properties and denaturation temperature of dentin collagen, making the collagen fibrils more resistant to degradation [24, 29, 30] and stabilizing the hybrid layer [28].

After crosslinking with 0.3 and 0.5 M carbodiimide, the enzyme activity was significantly inhibited, consistent with the findings of Scheffel [31]. The third null hypothesis was also rejected. Carbodiimide nonspecifically crosslinks all proteins by activating the carboxyl groups of glutamic and aspartic acids and then reacting with the ε-amino groups present in protein molecules, resulting in the creation of covalent cross-links [32]. Crosslinking increases the mechanical properties of dentin collagen and makes the fibrils more resistant to degradation [33]. In addition, carbodiimide crosslinks the peptide chains of proteases in the dentin matrix, irreversibly changing their three-dimensional structures and decreasing the molecular mobility of the catalytic sites of these enzymes, which are critical for their function [8, 34]. The present carbodiimide treatment combined with a self-etch adhesive was proved to be a feasible method in our in-vitro study. Clinical trials are necessary to confirm its effectiveness in maintaining dentin bonding durability. The clinical application of adding carbodiimide directly to the adhesive may simplify the bonding procedure which could be more appealing in dental clinic which could be explored in the further study.

With the limitations of this in vitro study, the following conclusions were drawn. Treatment with 0.3 or 0.5 M carbodiimide for 60 s did not reduce the immediate shear bond strength and delayed the decrease in shear bond strength after 12 months of water-storage aging, possibly due to increased crosslinking, enhanced thermal stability, and substantially decreased enzyme activity of dentin collagen. Applying carbodiimide combined with a self-etch adhesive may delay dentin bond aging.

## Data Availability

The datasets used and/or analysed during the current study available from the corresponding author on reasonable request.
